# Psychoanalysis and Neuroscience – A Disclosure

**DOI:** 10.3389/fnbeh.2018.00265

**Published:** 2018-11-06

**Authors:** Richard Brockman

**Affiliations:** Department of Psychiatry, College of Physicians and Surgeons, Columbia University, New York, NY, United States

**Keywords:** neuroscience, Freud, metapsychology, data collection, hypothesis

Disclosure.

I'm staring at the ceiling. I don't like this office. It's always felt small. It's always felt white. I stare at a shoe, polished and brown, dangling to my right.

“What are you thinking?”

He takes a drag on the cigarette.

Smoke. I'm thinking about smoke.

He sits in a chair as dark as his shoe.

“I had this dream. I'm skiing down a double black diamond. Six inches of powder. I cut a series of perfect turns. There is a crowd of people watching. They are impressed with my skill.”

I say, not so much to Dr. P. as to the wall on my left, that I miss the thrill of competitive skiing. I was once a really good skier -

“Six inches?” he asks.

- I still am. “Yeah, just enough to lay perfect tracks.”

The windows face onto the park. The room could be light and airy. Instead he keeps it dark and claustrophobic. I once met my once high school sweetheart in the waiting room. She was in treatment with Dr. P's colleague on the other side of the waiting room wall, a bald psychoanalyst, named Dr. Q.

“What are you thinking?”

I'm thinking about the waiting room meeting with my once high school sweetheart with whom I had shared an awkward silence, each of us thinking, “Oh God what are you doing here?” My once high school sweetheart's name is/was Vivian. Vivian K.

In the dream, the wind picks up, blowing snow in my eyes.

“Dreams are never concerned with trivia,” I remember reading somewhere in *The Interpretation of Dreams* (Freud, 1900).

“What are you thinking?”, he asks interrupting my thinking.

“I'm thinking about dreams,” I say. And then add, “And what they mean.”

“Dreams satisfy wishes,” again somewhere in *The Interpretation of Dreams*. Analysts I would learn, refer to the *Interpretation of Dreams* as “Chapter 7.” It's code.

Dr. P. clears his throat, then in his soft, strangely un-comforting tone, “The dream is about your desire to lay perfect tracks, a metaphor for sex, and the thought that your ‘tracks' are better than your father's.”

My father was raised on a shtetl in Poland. There was no skiing in or near the shtetl where he lived.

“What are you thinking?”

I'm thinking about dreams, perfect tracks, metaphors for sex.

On November 29th, 1895, Freud wrote to his friend and fellow physician, Wilhelm Fliess, “I no longer understand the state of mind in which I hatched the psychology; cannot conceive how I could have inflicted it on you.” He was referring to a manuscript, *The Project for a Scientific Psychology*, that he had sent to Fliess. By abandoning *The Project*, Freud was opening the door to Chapter 7. “It appears to have been a kind of madness,” again in that letter to Fliess (Freud, 1887–1905/1985, p. 152).

“What are you thinking?” he asks as he takes another drag on his cigarette.

The smoke drifts over my head.

He coughs.

I sit up.

“The hour is not over,” he says.

I swing my feet to the floor.

“Please lie back down.”

I don't say a word and then stand. “The hour is not over,” I hear again as I walk past Dr. P. who stays seated in his dark as shoe leather chair. I open the double inner doors, pass through the waiting room where I had once seen my once high school sweetheart Vivian, Vivian K (perhaps she had changed the hour of her appointment to avoid ever again overlapping with mine), out the outer door to the hall, down the elevator where the elevator operator always hummed one song or another as he took me up and down, this time down, a song by a reggae composer whose name I can't quite recall. To the ground. Out the building's door. Into air. City air.

What are you thinking?

“Gentlemen,” Freud began in a report he gave to the Medical Society of Vienna in April of 1896, “when we set out to form an opinion about the causation of a pathological state…”

In that report, Freud revealed that of 18 patients whom he had treated for “hysteria,” all 18 were found to have suffered from some form of sexual abuse during childhood, sometimes during the earliest years of childhood. From this data, Freud developed a therapeutic technique, “When we set out to form an opinion about the causation of a pathological state such as hysteria, we begin by adopting the method of anamnestic investigation” (Freud, 1896b, p.191).

He was describing a technique that he had learned from a mentor, Josef Breuer, that followed the path from symptoms to memory to treatment -

“Where shall we get if we follow the chains of associated memories which the analysis has uncovered? How far do they extend? Do they anywhere come to a natural end?”

From this beginning, the idea emerged that neurological illness (“hysteria” was then considered neurologic) could be caused by childhood sexual trauma that would not manifest itself until the victim had matured to such time as she would be able to more fully understand what had been done to her. The ultimate union of memory with affect, against the force of resistance, was this “natural end,”

“One only succeeds in awakening the psychical trace of a precocious sexual event under the most energetic pressure of the analytic procedure, and against the most enormous resistance,” (Freud, 1887–1905/1985, p. 153).

Everything about this was revolutionary. That psychological events could cause physical symptoms. That current symptoms could be related back to sexual assaults. That these assaults had occurred when the victim was a child. That these events were most often only partially understood and retained as fragments of memory. And that by following the chain of associations back from the inciting event that brought on the illness to the true etiologic event that had occurred to the child, a psychological method (psychoanalysis) was devised that could bring the fragments of memory and affect together into a coherent narrative and effect a cure. “Traveling backwards into the patient's past, step by step…I finally reached the starting-point of the pathological process” (Freud, 1896a, p.151). Everything about this was revolutionary.

Yet all of this would be abandoned on September 21st, 1897, when Freud wrote in another letter to Fliess, “I no longer believe in my *neurotica*” (Freud, 1887–1905/1985, p. 264). As of the date of this letter (actually somewhat before), Freud stopped believing that actual childhood seduction was the primary cause of hysteria.

A great deal has been made of this reversal.

It has been argued that Freud's abandonment of the “seduction theory” was a “failure of courage” (Masson, 1984, p. 19). That it reflected his desire to get back into the good graces of the Vienna Medical Society that had received his seduction theory with disapproving silence –

“A void is forming around me….my consulting room is empty” (Freud, 1887–1905/1985, p.185). That it reflected Freud's desire to protect his friend Wilhelm Fliess from accusations of incompetence after Fliess' operation on the nasal turbinates of Emma Eckstein nearly killed her (Freud would write to Fliess that the patient's post-operative near fatal hemorrhages were due to psychological factors, thus shifting blame from Fliess's malpractice to the patient's neurosis: “There is no doubt that her hemorrhages were due to wishes,” Freud wrote [Freud, 1887–1905/1985, p. 191]).

I do not believe that any of these really explain why Freud abandoned his *neurotica*. Rather I believe that Freud abandoned his theory of childhood seduction because he was not looking for the cause of a neurosis. Rather he was looking for the cause underlying all neurosis. He was looking for something he had sought in *The Project*. “The intention is to furnish a psychology that shall be a natural science” (Freud, 1895/1950, p. 295). But when he realized that he could not find the unifying principle in the brain, he sought to find the unifying principle in the mind. And he was confident he would.

“For I am actually not at all a man of science…I am by temperament nothing but a conquistador – an adventurer, if you want it translated – with all the curiosity, daring, and tenacity characteristic of a man of this sort. Such people are customarily esteemed only if they have been successful, have really discovered something” (Freud, 1887–1905/1985, p. 398).

Freud abandoned the seduction theory not because he felt it wasn't valid, but because he realized it wasn't universal. He made this discovery when he uncovered an error in his formulation, “…that in all cases, the *father*, not excluding my own, had to be accused of being perverse….” (Freud, 1887–1905/1985, p. 264). From this error, Freud was lead to another more subtle error: “I attributed to the aetiological factor of seduction a significance and universality which it does not possess” (Freud, 1896c, p. 168 footnote 1, 1924). And from this realization, he knew he had to look elsewhere for the universal. And to find it, he turned to a normal mind, to his own—“My self-analysis is in fact the most essential thing….” (Freud, 1887–1905/1985, p.270).

From that point, it did not take Freud long to discover the universal that he had sought. Indeed 3 weeks after writing to Fliess exclaiming that he was lost (September 21st, 1897), he wrote that he was found (October 15, 1897).

“A single idea of general value dawned on me. I have found, in my case too, being in love with my mother and jealous of my father, and I now consider it a universal event in early childhood…” (Freud, 1887–1905/1985, p. 272).

By studying his own mind, Freud discovered that the universal factor was not actual seduction of the child. The universal factor was the child's desire to be seduced. And thus, less than a year after having discovered what he came to regard as a false source of a neurosis in particular—“*one or more occurrences of premature sexual experience*” (Freud, 1896b, p. 203)—he discovered the source of neurosis in general—*infantile sexual phantasy*. And like the image of the conquistador he so admired, Freud was now certain he had “really discovered something,” something that was common to every man, woman and child –

“We can understand the gripping power of *Oedipus Rex*…Everyone in the audience was a budding Oedipus in fantasy and each recoils in horror from the dream fulfillment here transplanted into reality, with the full quantity of repression which separates his infantile state from his present one” (Freud, 1887–1905/1985, p. 272).

Some have argued, including his daughter, Anna, that Freud had to sacrifice the seduction theory in order for psychoanalysis to be born,

“Keeping up the seduction theory would mean to abandon the Oedipus complex, and with it the whole importance of phantasy life, conscious or unconscious phantasy. In fact, I think there would have been no psychoanalysis afterwards” (September 10, 1981 in Malcolm, 1983, p. 63).

And thus the universal agent at the heart of neurosis, was revealed –

“If hysterical subjects trace back their symptoms to traumas that are fictitious, then the new fact which emerges is precisely that they create such scenes in *phantasy*.” (Freud, 1914, p. 17).

- the universal agent that would be at the very foundation of psychoanalysis.

But there was a hurdle. If a child's fantasies of seduction were at least as powerful as actual experiences of childhood seduction, rape, and/or violence, then the newly discovered power of fantasy would have to be explained. And it was, by another fact:

“It remains a fact that the patient has created these phantasies for himself, and this fact is of scarcely less importance for his neurosis than if he had really experienced what the phantasies contain. The phantasies possess *psychical* as contrasted with *material* reality, and we gradually learn to understand that *in the world of the neuroses it is psychical reality which is the decisive kind*” (Freud, 1916–1917, p. 368, italics in original).

This new fact established fantasy to be as powerful as reality because in the world of the neuroses it is psychical reality which is the decisive kind.

Freud needed to make this leap in order to explain the power of fantasy. It was with this leap that he was able to explain how fantasy could create illness. It was with this leap that he established the science of psychoanalysis. And it was with this leap where things between me and Freud got personal -

“What are you thinking?”

- because several months after beginning treatment with Dr. P., I encountered my once high school sweetheart in the shared waiting room of Drs. P and Q. I don't know what my once high school sweetheart said to Dr. Q. I don't know what Dr. Q. said to her because I never saw her again. I just know that 5 years after seeing my once high school sweetheart that one time in that shared waiting room, she committed suicide.
- because when I was a boy, my mother was sent to see a psychoanalyst, Dr. S. I don't know why she was sent to see Dr. S. I don't know what she said to Dr. S. I don't know what Dr. S. said to her. I just know that when I was seven years, 2 months and 2 days old, my mother walked down the stairs to the basement of our house in the Sheepshead Bay section of Brooklyn and hung herself.

Part of me always thought that my one encounter with my once high school sweetheart in the waiting room of Drs. P. and Q. had in some way contributed to her death. Even though it was 5 years later when she killed herself. Even though it was she who broke up with me. Even though my once high school sweetheart and I had never had sex. Even though I still wanted to when I saw her in the waiting room of Drs. P. and Q. Even though I never mentioned any of this. Not to her. Not to him. Maybe that's what the dream was about. Sex and love and a once high school sweetheart and death.

Part of me always felt responsible for the death of my mother. Part of me always felt that her death was my fault. Part of me always felt I should have saved her. Even though I had no idea that anything had been wrong. Even though I was seven years, 2 months, and 2 days old. Even though I loved her as deeply as any child could and still do.

“What are you thinking?”

I'm thinking it was Freud, not me, who contributed to the death of my once high school sweetheart. I'm thinking it was Freud, not me, who contributed to the death of my mother. I'm thinking is was Freud not me who caused harm to people I loved. I'm thinking it was Freud—that's what I've been thinking. And I've been thinking that I'm not sure it's fair to blame any or all of this on Freud. Or on myself. But I do.

Is it fair to blame psychoanalysis for their suicides?

Is it fair to blame myself for their suicides?

I don't know.

I just know this is personal.

I became a physician to become a psychiatrist. I became a psychiatrist to become a psychoanalyst. I went to analytic school. I felt the only way for me to save my mother and my once high school sweetheart was to become one of those who in my mind, had killed them. It was a fantasy of rescue. It was a fantasy of revenge. The fantasy was “overdetermined” in the parlance of psychoanalysis. But I never became a psychoanalyst. I quit psychoanalytic school the way I quit Dr. P. I just left.

But I studied psychoanalysis. I learned its teachings. I learned its codes. And thus this paper, this confession, this disclosure is an “inside job.”

If psychoanalysis is to survive, it must accomplish what Freud set out to do when he started *The Project*. If psychoanalysis is to survive it must rid itself of every hypothesis founded on antecedent hypothesis. If psychoanalysis is to survive, it must never allow one of its own to say to someone like me, that the dream reflects a desire to lay “sexual” tracks better than my father's or some other blurred Oedipal crap. If psychoanalysis is to survive, it must never allow anyone to repeat what was done to my once high school sweetheart. If psychoanalysis is to survive, it must never allow anyone to repeat what was done to my mother. If psychoanalysis is to survive, it must never describe anything Freud wrote after 1897 (or anything derived from what he wrote after 1897) as “science.” If psychoanalysis is to survive it must never call on neuroscience to justify its “facts.” If psychoanalysis is to survive it must be honest with itself.

And if psychoanalysis can't be honest with itself, then it shouldn't survive. If psychoanalysis can't be honest with itself, then I will do everything in my power to destroy it.

But if psychoanalysis is to survive, then it must sacrifice many if not most of its most cherished “facts” because almost all of psychoanalysis after 1897 was derived from a core hypothesis that had incubated in Freud's mind from sometime in early 1896 when he abandoned the *Project*, until that day in September 1897 when new insight dawned. It was a hypothesis that was brilliant, compelling, persuasive—the insight of a conquistador, the kind of insight that comes “but once in a lifetime.” And it was wrong. Dead wrong.

This key insight, “The certain conviction of the existence and importance of infantile sexuality…” (Freud, 1914, p. 18) lead Freud to the awareness of repression: “We have learnt from psycho-analysis that the essence of the process of repression lies, not in putting an end to…the idea which represents an instinct, but in preventing it from becoming conscious.” From this he was lead to discover the unconscious, “When this happens, we say of the idea that it is in a state of being ‘unconscious”' (Freud, 1915, p. 166). And thus Freud established the fact of *infantile sexual phantasy* by explaining that it was buried deep in the unconscious and kept there by the force of repression. Because of repression, the only way to become aware of *infantile sexual phantasy*, is via the method that Freud had developed, “The certain conviction of the existence and importance of infantile sexuality, can, however, only be obtained by the method of analysis …” (Freud, 1914, p. 18).

The implication, of course, is that if one has failed to uncover *infantile sexual phantasy* in one's analysis, it is not because such fantasies were not there, rather it is because the analysis itself failed or because the repression was too powerful. Either way, there was never any doubt of the existence of these factors, “There are two positions which I have never repudiated or abandoned – the importance of sexuality and of infantilism.” (Freud, 1906, p. 278).

“This is probably not intelligible without an explanation” (Freud, 1887–1905/1985, p. 264) Freud wrote in that September letter.

Between early 1896, when he abandoned *The Project*, and September 21st, 1897 when he abandoned his *neurotica*, Freud's thinking went through a gradual but ultimately radical change. His thinking went from the hypothesis that childhood sexual trauma was the basis for hysterical illness in particular, to the hypothesis that childhood fantasy was the basis for neurotic illness in general. It wasn't that trauma wasn't a factor for some. It was that fantasy was a factor for all. It was a shift from external reality to internal instinct. It was a shift from the biology of brain to the psychology of mind. And it was a shift from the methods of science to the methods of psychoanalysis.

And because all data was now the data of psychoanalytic observation, Freud treated his clinical observations as though they had the rigor of science. Plausible speculation became fact. Persuasive argument, proof. The posing of a question established the assumptions that underlay the question. And so when Freud asked, “Whence comes the need for these phantasies and the material for them?”—it was as if the question had transformed a clinical hypothesis into a scientific fact—as if the question about infantile sexual fantasy had established the fact of *infantile sexuality phantasy*. And so from the question—“Whence comes the need for these phantasies and the material for them?”—came a response that not only explained but also confirmed their existence: “There can be no doubt that their sources lie in the instincts…”

And having established their existence as derived from an “instinct,” Freud then went on to provide the history of their origin, “I am prepared with an answer which I know will seem daring to you. I believe these *primal phantasies*…are a phylogenetic endowment. In them the individual reaches beyond his own experience into primeval experience at points where his own experience has been too rudimentary.”

And having established this origin, Freud then needed to explain just how *infantile sexual phantasies* of violence and seduction that are recreated in psychoanalytic transference and dream, have the power of actual violence and seduction. They have this power, he explained, because even if they are fantasies now, they were once real.

“It seems to me quite possible that all the things that are told to us to-day in analysis as phantasy – the seduction of children, the inflaming of sexual excitement by observing parental intercourse, the threat of castration (or rather castration itself) - were once real occurrences in the primeval times of the human family, and that children in their phantasies are simply filling in gaps in individual truth with prehistoric truth” (Freud, 1916–1917p. 370–371).

And so not only is the existence of infantile sexual fantasy established, but the incredible power of infantile sexual fantasy is also established by this “phylogenic endowment.” Fantasy thus has the power of reality because it once was real. And so a hypothesis about the power of *infantile sexual phantasy* has become fact. As has the instinct. As has the endowment. And because of these facts, fantasy has the force of reality.

“When I had pulled myself together I was able to draw the right conclusions from my discovery: namely that the neurotic symptoms were not related directly to actual events but to wishful phantasies, and that as far as the neurosis was concerned psychical reality was of more importance than material reality” (Freud, 1925, p. 34).

Once Freud had made his discovery, no data was needed to establish the validity of *infantile sexual phantasy*. Phylogenic endowment established fantasies in the infant's mind. Repression kept them out of awareness in the unconscious. Psychoanalysis demonstrated this fact.

Freud was so convinced of the validity of *infantile sexual phantasy* (a fact that he confirmed in his self-analysis) that his actual observation of an infant was unnecessary. “Why do I not go into the nursery and experiment with Annerl?” he asked in a letter to Fliess referring to his then 2-year-old daughter, Anna.

Darwin, unlike Freud, had spent quite a bit of time playing with and observing his children. “I attended to this point in my first-born infant…I was convinced that he understood a smile and received pleasure from seeing one, answering it by another, at much too early an age to have learnt anything by experience” (Darwin, 1872/1965, p. 358). Darwin had thus observed that his son was instinctively responsive to his environment pretty much from birth. Freud wrote in the margins of his copy of the *Expression of the Emotions in Man and Animals*, the book from which this quote of Darwin's is taken. So there is no question but that Freud was aware of Darwin's observations. But Freud was apparently not impressed. He did not feel observations in the nursery were necessary. Or at least, he wrote, “I have no time for it” (Freud, 1887–1905/1985, p. 230).

And so Freud extended his argument. He theorized that an infant is born with *infantile sexual phantasy* active at the very moment of birth. For this reason, the neonate does not seek his/her mother. The neonate instead seeks pleasure from *auto-eroticism*. Only after the failure of *auto-eroticism* (the failure of the Freudian primary process) does the neonate realize that fantasy is failing to provide pleasure (nourishment), and then seeks a remedy (the mother). “The process of arriving at an *object…*takes place alongside of the organization of the libido.” The mother is not the neonate's first choice. Because it is only “After the stage of *auto-eroticism*, (that) the first love-object in the case of both sexes is the mother; and it seems probable that to begin with a child does not distinguish its mother's organ of nutrition from its own body” (Freud, 1925, p. 36). Thus, *auto-eroticism* (another expression for *infantile sexual phantasy*) is our first consciousness. Only after the neonate realizes that in order to find nutrition it must find another, does *auto-eroticism* and the pleasure principle give way to the search for the mother and the reality principle.

Freud's response as to why he didn't spend some more time with his daughter, Annerl, may have been less a fact, than that in order for Freud to maintain the idea that he had discovered the universal principle of neurosis, he needed to argue that not only was repression the corner stone of psychoanalysis (“The theory of repression is the corner-stone on which the whole structure of psycho-analysis rests” [Freud, 1914, p. 16]), but most critically that *infantile sexual phantasy* was the first content of mind. In other words, once Freud had established *infantile sexual phantasy* as universal, then everything had to follow from that.

Looking to the future, Freud had two quite different takes on how his ideas would be viewed. In (Freud, 1914), he wrote: “Science would ignore me entirely during my lifetime; some decades later, someone else would infallibly come upon the same things…would achieve recognition for them and bring me honor as a forerunner whose failure had been inevitable” (p. 22).

Six years later in 1920, his sense of how he would 1 day be received had changed. It was as if he were returning to the bolder, scientific vision he had when he began to write *The Project*,

“Biology is truly a land of unlimited possibilities. We may expect it to give us the most surprising information, and we cannot guess what answers it will return in a few dozen years to the questions we have put to it. They may be of a kind which will blow away the whole of our artificial structure of hypotheses” (p. 60).

If neuroscience seeks to answer some of the questions raised by psychoanalysis, then it should take this (Freud, 1920) statement of Freud's as his “wish” and “blow away the whole of (his) artificial structure of hypotheses.” Because his artificial structure of hypotheses is beautiful, compelling, convincing, and dangerous. It contributed to the deaths of two people I loved.

Disclosure:

I blame Freud. (I am not the first to find fault with Freud's a-scientific theories. Jeffrey Masson, Janet Malcom, John Bowlby—there are of course many more).

Disclosure:

I was never analyzed. I got up off the couch that last time, opened inner doors, walked through the waiting room where I had once encountered my once high school sweetheart, moved through the hall to the elevator where the elevator man was humming a song by a reggae composer whose name I just can't quite –

Marley.

He was humming a song by Robert Marley.

## Author contributions

The author confirms being the sole contributor of this work and has approved it for publication.

### Conflict of interest statement

The author declares that the research was conducted in the absence of any commercial or financial relationships that could be construed as a potential conflict of interest.

## References

[B1] Darwin C. (1872/1965) The Expression of the Emotions in Man and Animals. Chicago, IL: University of Chicago Press.

[B2] Freud S. (1916–1917). Introductory lectures on psychoanalysis, in The Standard Edition of the Complete Psychological Works of Sigmund Freud, Vol. 16, Transl. by Strachey J. (London: Hogarth Press), 243–463.

[B3] Freud S. (1887–1905/1985). The Complete Letters of Sigmund Freud to Wilhelm Fliess. Transl. by Masson J. M. London: Belknap Press.

[B4] Freud S. (1895/1950). Project for a scientific psychology, in The Standard Edition of the Complete Psychological Works of Sigmund Freud, Vol. 1, Transl. by Strachey J. (London: Hogarth Press), 295–397.

[B5] Freud S. (1896a). Heredity and the neuroses, in The Standard Edition of the Complete Psychological Works of Sigmund Freud, Vol. 3, Transl. by Strachey J. (London: Hogarth Press), 143–156.

[B6] Freud S. (1896b). The aetiology of hysteria, in The Standard Edition of the Complete Psychological Works of Sigmund Freud, Vol. 3, Transl. by Strachey J. (London: Hogarth Press), 189–221.

[B7] Freud S. (1896c). Further remarks on the neuropsychoses of defense, in The Standard Edition of the Complete Psychological Works of Sigmund Freud, Vol. 3, Transl. by Strachey J. (London: Hogarth Press), 163–185.

[B8] Freud S. (1900). The interpretation of dreams, in The Standard Edition of the Complete Psychological Works of Sigmund Freud, Vol. 5, Transl. by Strachey J. (London: Hogarth Press), 339–685.

[B9] Freud S. (1906). Sexuality in the neuroses, in The Standard Edition of the Complete Psychological Works of Sigmund Freud, Vol. 7, Transl. by Strachey J. (London: Hogarth Press), 271–279.

[B10] Freud S. (1914). On the history of the psychoanalytic movement, in The Standard Edition of the Complete Psychological Works of Sigmund Freud, Vol. 14, Transl. by Strachey J. (London: Hogarth Press), 7–66.

[B11] Freud S. (1915). The unconscious, in The Standard Edition of the Complete Psychological Works of Sigmund Freud, Vol. 14, Transl. by Strachey J. (London: Hogarth Press), 7–66.

[B12] Freud S. (1920). Beyond the pleasure principle, in The Standard Edition of the Complete Psychological Works of Sigmund Freud, Vol. 18, Transl. by Strachey J. (London: Hogarth Press), 7–64.

[B13] Freud S. (1925). An autobiographical study, in The Standard Edition of the Complete Psychological Works of Sigmund Freud, Vol. 20, Transl. by Strachey J. (London: Hogarth Press), 7–74.

[B14] Malcolm J. (1983). In the Freud Archives. New York, NY: New York Review Books.

[B15] Masson J. M. (1984). The Assault on Truth. New York, NY: Pocket Books.

